# Glucagon-Like Peptide-1 Excites Firing and Increases GABAergic Miniature Postsynaptic Currents (mPSCs) in Gonadotropin-Releasing Hormone (GnRH) Neurons of the Male Mice via Activation of Nitric Oxide (NO) and Suppression of Endocannabinoid Signaling Pathways

**DOI:** 10.3389/fncel.2016.00214

**Published:** 2016-09-12

**Authors:** Imre Farkas, Csaba Vastagh, Erzsébet Farkas, Flóra Bálint, Katalin Skrapits, Erik Hrabovszky, Csaba Fekete, Zsolt Liposits

**Affiliations:** ^1^Laboratory of Endocrine Neurobiology, Institute of Experimental Medicine, Hungarian Academy of SciencesBudapest, Hungary; ^2^Laboratory of Integrative Neuroendocrinology, Institute of Experimental Medicine, Hungarian Academy of SciencesBudapest, Hungary; ^3^Roska Tamás Doctoral School of Sciences and Technology, Faculty of Information Technology and Bionics, Pázmány Péter Catholic UniversityBudapest, Hungary; ^4^Department of Medicine, Division of Endocrinology, Diabetes and Metabolism, Tupper Research Institute, Tufts Medical CenterBoston, MA, USA; ^5^Department of Neuroscience, Faculty of Information Technology and Bionics, Pázmány Péter Catholic UniversityBudapest, Hungary

**Keywords:** GnRH neuron, GABA, glucagon-like peptide-1, endocannabinoid, nitric oxide, retrograde signaling, TRPV1, anandamide

## Abstract

Glucagon-like peptide-1 (GLP-1), a metabolic signal molecule, regulates reproduction, although, the involved molecular mechanisms have not been elucidated, yet. Therefore, responsiveness of gonadotropin-releasing hormone (GnRH) neurons to the GLP-1 analog Exendin-4 and elucidation of molecular pathways acting downstream to the GLP-1 receptor (GLP-1R) have been challenged. Loose patch-clamp recordings revealed that Exendin-4 (100 nM–5 μM) elevated firing rate in hypothalamic GnRH-GFP neurons of male mice via activation of GLP-1R. Whole-cell patch-clamp measurements demonstrated increased excitatory GABAergic miniature postsynaptic currents (mPSCs) frequency after Exendin-4 administration, which was eliminated by the GLP-1R antagonist Exendin-3(9–39) (1 μM). Intracellular application of the G-protein inhibitor GDP-β-S (2 mM) impeded action of Exendin-4 on mPSCs, suggesting direct excitatory action of GLP-1 on GnRH neurons. Blockade of nitric-oxide (NO) synthesis by Nω-Nitro-L-arginine methyl ester hydrochloride (L-NAME; 100 μM) or N^5^-[Imino(propylamino)methyl]-L-ornithine hydrochloride (NPLA; 1 μM) or intracellular scavenging of NO by 2-(4-carboxyphenyl)-4,4,5,5-tetramethylimidazoline-1-oxyl-3-oxide (CPTIO; 1 mM) partially attenuated the excitatory effect of Exendin-4. Similar partial inhibition was achieved by hindering endocannabinoid pathway using cannabinoid receptor type-1 (CB1) inverse-agonist 1-(2,4-dichlorophenyl)-5-(4-iodophenyl)-4-methyl-N-(1-piperidyl) pyrazole-3-carboxamide (AM251; 1 μM). Simultaneous blockade of NO and endocannabinoid signaling mechanisms eliminated action of Exendin-4 suggesting involvement of both retrograde machineries. Intracellular application of the transient receptor potential vanilloid 1 (TRPV1)-antagonist 2E-N-(2, 3-Dihydro-1,4-benzodioxin-6-yl)-3-[4-(1, 1-dimethylethyl)phenyl]-2-Propenamide (AMG9810; 10 μM) or the fatty acid amide hydrolase (FAAH)-inhibitor PF3845 (5 μM) impeded the GLP-1-triggered endocannabinoid pathway indicating an anandamide-TRPV1-sensitive control of 2-arachidonoylglycerol (2-AG) production. Furthermore, GLP-1 immunoreactive (IR) axons innervated GnRH neurons in the hypothalamus suggesting that GLP-1 of both peripheral and neuronal sources can modulate GnRH neurons. RT-qPCR study confirmed the expression of GLP-1R and neuronal NO synthase (nNOS) mRNAs in GnRH-GFP neurons. Immuno-electron microscopic analysis revealed the presence of nNOS protein in GnRH neurons. These results indicate that GLP-1 exerts direct facilitatory actions via GLP-1R on GnRH neurons and modulates NO and 2-AG retrograde signaling mechanisms that control the presynaptic excitatory GABAergic inputs to GnRH neurons.

## Introduction

Glucagon-like peptide-1 (GLP-1) is one of the metabolic signal molecules, discovered in humans in 1987 (Kreymann et al., 1987). It is secreted by the intestinal L-cells as a gut hormone (Mojsov et al., 1990; Baggio and Drucker, 2007). It reduces food intake (Kinzig et al., 2002; Hayes et al., 2008), inhibits gastric emptying (Chelikani et al., 2005), and increases glucose-stimulated insulin secretion (Komatsu et al., 1989; Drucker, 1998; Knauf et al., 2005). Radiolabeled GLP-1 and GLP-1 receptor (GLP-1R) agonists can cross the blood-brain barrier (Hunter and Hölscher, 2012), suggesting an ability to reach various control centers of homeostasis. In addition to the small intestine, GLP-1 is also produced in neurons of the lower brain stem, clustering in the nucleus of the solitary tract (NST) and the reticular nucleus of the medulla oblongata (Larsen et al., 1997; Merchenthaler et al., 1999; Vrang and Larsen, 2010). GLP-1 immunoreactive (IR) fibers and terminals were observed in the hypothalamus, thalamus, septal regions, cortex and hindbrain as reviewed in Trapp and Cork (2015). GLP-1R is widely expressed in the human and rodent brains such as in neurons of the circumventricular organs, amygdala, hypothalamic nuclei, ventrolateral medulla, NST, thalamic paraventricular nucleus, hippocampus and cortex, in various loci for hypothalamic regulation of glucose homeostasis, and parabrachial nucleus a regulatory center of feeding behavior (Goke et al., 1995; Wei and Mojsov, 1995; Scrocchi et al., 1996; Li et al., 2003; Sandoval et al., 2008; Richard et al., 2014; Richards et al., 2014; Cork et al., 2015; Sandoval and Sisley, 2015).

In addition to regulating energy homeostasis, GLP-1 is a potent regulator of reproduction. Accordingly, its intracerebroventricular injection resulted in an increase in the plasma luteinizing hormone (LH) level of male rats, and GLP-1 elicited a concentration-dependent increase in gonadotropin-releasing hormone (GnRH) release from the immortalized GnRH-producing GT1–7 neurons (Beak et al., 1998). Male GLP-1R knockout mice exhibited reduced gonadal weights, and females possessed a slight delay in the onset of puberty (MacLusky et al., 2000). GLP-1 doubled the amplitude of the preovulatory LH surge, influenced estradiol and progesterone levels and increased the number of Graafian follicles and corpora lutea (Outeiriño-Iglesias et al., 2015). Since GnRH neurons orchestrate the hypothalamo-pituitary-gonadal axis, any GLP-1-induced modulation of the GnRH neuronal system itself has a major impact on various events of reproductive physiology. Although some of the intracellular elements of the GLP-1 activated pathway such as an elevated cytoplasmic cAMP level has already been identified in the GT1–7 cells (Beak et al., 1998), the exact target and detailed molecular mechanism involved in the downstream actions of GLP-1 in GnRH neurons of the rodent preoptic area have not been elucidated, yet.

Therefore, the present study was aimed at revealing the putative direct effects of GLP-1 upon electric activity of GnRH neurons in male mice. Patch-clamp recordings in GnRH-GFP neurons revealed GLP-1 induced changes in firing. Then, effects of GLP-1 in the GABAergic miniature postsynaptic currents (mPSCs) were examined, because GABA is the main excitatory neurotransmitter via GABA_A_-R in GnRH neurons (Moenter and DeFazio, 2005; Yin et al., 2008; Herbison and Moenter, 2011). GABAergic mPSCs in GnRH neurons are dampened by the tonic and triggered retrograde release of endocannabinoid 2-arachidonoylglycerol (2-AG; Farkas et al., 2010, 2013). In contrast, nitric oxide (NO) can facilitate postsynaptic currents in hypothalamic neurons (Di et al., 2009). Thus, in the GLP-1R-mediated actions the regulatory role of retrograde endocannabinoid and putative NO signaling pathways was addressed by tools of slice electrophysiology in GnRH neurons of mice. Immunocytochemical study was carried out to reveal whether axons containing GLP-1 innervate GnRH neurons. Immuno-electron microscopic analysis addressed the putative expression of neuronal NO synthase (nNOS) enzyme in GnRH neurons. Meanwhile, RT-qPCR analysis of individual GnRH neurons was used to substantiate the expression of GLP-1R and nNOS in mouse GnRH neurons.

## Materials and Methods

### Animals

Adult male mice were used from local colonies bred at the Medical Gene Technology Unit of the Institute of Experimental Medicine (IEM). They were housed in light (12:12 light-dark cycle, lights on at 06:00 h)—and temperature (22 ± 2°C) controlled environment, with free access to standard food and tap water. GnRH-green fluorescent protein (GnRH-GFP) transgenic mice (*n* = 70) bred on a C57Bl/6J genetic background were used for electrophysiological experiments. In this animal model, a GnRH promoter segment drives selective GFP expression in the majority of GnRH neurons (Suter et al., 2000). Experiments studying the presence of nNOS in GnRH neurons were carried out using C57Bl/6J mice and mice lacking nNOS (nNOS^−/−^) generated by the Jackson Laboratory (Bar Harbor, ME, USA; Szabadits et al., 2007).

### Ethics Statement

All animal studies were carried out with permissions from the Animal Welfare Committee of the IEM Hungarian Academy of Sciences (Permission Number: A5769-01) and in accordance with legal requirements of the European Community (Decree86/609/EEC). All animal experimentation described was conducted in accord with accepted standards of humane animal care and all efforts were made to minimize suffering. Sacrifice of animals for electrophysiological studies was carried out by decapitation in deep anesthesia by Isoflurane inhalation.

### Brain Slice Preparation and Recordings

Mice were deeply anesthetized using Isoflurane inhalation. The brain was removed rapidly and immersed in ice cold sodium-free artificial cerebrospinal fluid (Na-free aCSF) bubbled with a mixture of 95% O_2_ and 5% CO_2_. The solution contained the following (in mM): saccharose 205, KCl 2.5, NaHCO_3_ 26, MgCl_2_ 5, NaH_2_PO_4_ 1.25, CaCl_2_ 1, glucose 10. Hypothalamic blocks were dissected and 250 μm thick coronal slices were prepared from the medial septum/preoptic area with a Leica VT-1000S vibratome (Leica Microsystems, Wetzlar, Germany) in the ice-cold oxygenated Na-free aCSF. The slices were equilibrated in normal aCSF (in mM): NaCl 130, KCl 3.5, NaHCO_3_ 26, MgSO_4_ 1.2, NaH_2_PO_4_ 1.25, CaCl_2_ 2.5, glucose 10, saturated with O_2_/CO_2_ for 1 h. Initial temperature of aCSF was 33°C which was left to cool to room temperature during equilibration.

Recordings were carried out in oxygenated aCSF at 33°C. Axopatch-200B patch-clamp amplifier, Digidata-1322A data acquisition system, and pCLAMP 10.4 software (Molecular Devices Co., Silicon Valley, CA, USA) were used for recording. Cells were visualized with a BX51WI IR-DIC microscope (Olympus Co., Tokyo, Japan). The patch electrodes (OD = 1.5 mm, thin wall, Hilgenberg GmBH, Malsfeld, Germany) were pulled with a Flaming-Brown P-97 puller (Sutter Instrument Co., Novato, CA, USA) and polished with an MF-830 microforge (Narishige Inc., Tokyo, Japan).

GnRH-GFP neurons in the close proximity of the vascular organ of lamina terminalis (OVLT; Bregma 0.49–0.85 mm) were identified by brief illumination at 470 nm using an epifluorescent filter set, based on their green fluorescence, typical fusiform shape and characteristic topography (Suter et al., 2000).

Loose-patch or whole-cell patch-clamp measurements were carried out with an initial control recording (5 min), then Exendin-4 (100 nM–5 μM) or the NO-donor L-arginine (1 mM) was added to the aCSF in a single bolus onto the slice in the recording chamber and the recording continued for a subsequent 10 min. The GLP-1R antagonist Exendin-3(9–39) (1 μM), the NO-synthase (NOS) inhibitor Nω-Nitro-L-arginine methyl ester hydrochloride (L-NAME; 100 μM), the nNOS inhibitor N^5^-[Imino(propylamino)methyl]-L-ornithine hydrochloride (NPLA; 1 μM) or the cannabinoid receptor type-1 (CB1) inverse agonist AM251 (1 μM) were added to the aCSF 10 min before adding the Exendin-4 and they were then continuously present in the aCSF during the electrophysiological recording. Intracellularly applied drugs, such as the membrane impermeable G-protein inhibitor Guanosine 5′-[β;-thio] diphosphate (GDP-β-S; 2 mM), the membrane impermeable NO-scavenger 2-(4-carboxyphenyl)-4,4,5,5-tetramethylimidazoline-1-oxyl-3-oxide (CPTIO; 1 mM), the transient receptor potential vanilloid 1 (TRPV1) antagonist AMG9810 (10 μM), NPLA (1 μM), or the anandamide-degrading enzyme fatty acid amide hydrolase (FAAH) inhibitor PF3845 (5 μM) were added to the intracellular pipette solution and after achieving whole-cell patch clamp configuration, we waited 15 min to reach equilibrium in the intracellular milieu before starting recording. Each neuron served as its own control when drug effects were evaluated.

### Reagents and Chemicals

Exendin-4 (100 nM–5 μM, Tocris, Bristol, UK, Eng et al., 1992; Raufman et al., 1992; Acuna-Goycolea and van den Pol, 2004); NO-donor L-arginine (1 mM, Sigma, St. Louis, MO, USA, Makara et al., 2007; Lameu et al., 2012); GLP-1R antagonist Exendin-3(9–39) (1 μM, Tocris, Eng et al., 1992; Raufman et al., 1992; Acuna-Goycolea and van den Pol, 2004); NOS inhibitor L-NAME (100 μM; Sigma, Makara et al., 2007; Poglia et al., 2011); nNOS inhibitor NPLA (1 μM; Tocris, Chow et al., 2012; Filpa et al., 2015; Gong et al., 2015); CB1 inverse agonist 1-(2,4-dichlorophenyl)-5-(4-iodophenyl)-4-methyl-*N*-(1-piperidyl) pyrazole-3-carboxamide(AM251; 1 μM; Tocris, Farkas et al., 2010, 2013); G-protein inhibitor GDP-β-S (2 mM; Sigma, Meis et al., 2002; Ponzio and Hatton, 2005; McDermott and Schrader, 2011); NO-scavenger CPTIO (1 mM, Sigma, Makara et al., 2007; Mironov and Langohr, 2007); TRPV1 antagonist 2E-N-(2,3-Dihydro-1,4-benzodioxin-6-yl)-3-[4-(1,1-dimethylethyl)phenyl]-2-Propenamide (AMG9810; 10 μM; Sigma, Vriens et al., 2011; Liu and Zhuo, 2014; Jian et al., 2016); anandamide-degrading enzyme FAAH inhibitor PF3845 (N-3-Pyridinyl-4-[[3-[[5-(trifluoromethyl)-2-pyridinyl]oxy]phenyl]methyl]-1-piperidinecarboxamide hydrate; 5 μM; Sigma, Lee et al., 2015).

### Loose-Patch-Clamp Experiments

Loose-patch measurements were carried out to record action currents in GnRH-GFP neurons of the acute brain slice as described earlier (Farkas et al., 2010). Briefly, pipette potential was set to 0 mV, pipette resistance was 1–2 MΩ, resistance of loose-patch seal 7–40 MΩ. The pipette solution contained (in mM): NaCl 150, KCl 3.5, CaCl_2_ 2.5, MgCl_2_ 1.3, HEPES 10, glucose 10 (pH = 7.3 with NaOH).

### Whole-Cell Patch-Clamp Experiments

Resting potential (V_rest_) was recorded in current-clamp. The mPSCs in GnRH neurons were measured as described earlier (Farkas et al., 2010). Briefly, the neurons were voltage-clamped at –70 mV holding potential. Intracellular pipette solution contained (in mM): HEPES 10, KCl 140, EGTA 5, CaCl_2_ 0.1, Mg-ATP 4, Na-GTP 0.4 (pH = 7.3 with NaOH). The resistance of the patch electrodes was 2–3 MΩ. Spike-mediated transmitter release was blocked in all experiments by adding the voltage-sensitive Na-channel inhibitor tetrodotoxin (TTX, 660 nM, Tocris) to the aCSF 10 min before mPSCs or V_rest_ were recorded. The mPSCs recorded under the conditions used in our experiments were related to GABA_A_-R activation (Sullivan et al., 2003; Farkas et al., 2010). This GABAergic input via GABA_A_-R is excitatory to GnRH cells (Moenter and DeFazio, 2005; Yin et al., 2008; Herbison and Moenter, 2011), although we have to note that GABA inhibits GnRH neurons via GABA_B_-receptors (GABA_B_-R; Herbison and Moenter, 2011; Liu and Herbison, 2011).

### GLP-1 and GnRH Double Immunofluorescent Studies in the Mouse Hypothalamus

Adult male mice were deeply anesthetized and perfused transcardially with 4% paraformaldehyde (in 0.05 M PBS, pH = 7.4). Brains were removed from the skulls, postfixed for 30 min, and then equilibrated in 20% sucrose-Phosphate-buffered saline (PBS) for cryoprotection. Twenty-five μm thick serial coronal sections were cut with a cryostat (Leica CM3050; Leica Biosystems Nussloch GmbH, Nussloch, Germany). Sections were washed several times in PBS followed by antigen retrieval in 0.01 M sodium-citrate (pH = 6) at 80°C for 30 min, then permeabilized with 0.5% Triton-X100 for 20 min and blocked in 2% normal goat serum in PBS for 1 h at room temperature. The specificity of the primary antisera used in this experiment was validated earlier (Theodorakis et al., 2006; Gautron et al., 2011; Hrabovszky et al., 2011). Polyclonal antibodies raised in guinea pig against GnRH (EH#1018; 1:5000; 48 h at 4°C) were reacted with FITC conjugated anti-guinea pig IgG (1:500; Jackson ImmunoResearch Laboratories). The rabbit polyclonal GLP-1 antiserum (#T-4057; 1:2000; Peninsula Labs, San Carlos CA, USA) was applied to the sections for 48 h at 4°C, then reacted with biotinylated anti rabbit-IgG (Jackson; 1:500 in PBS for 2 h at room temperature), followed by Streptavidin-Cy3 (Jackson; 1:1000). Sections were mounted on glass slides, air-dried and coverslipped in Mowiol (Calbiochem, San Diego, CA, USA). Images were acquired by a Nikon C2 confocal microscope (Nikon Corp., Tokyo, Japan).

### Double-Labeling Immuno-Electron Microscopy for nNOS and GnRH

Wild type and nNOS^−/−^ mice were anesthetized with a mixture of ketamine and xylazine (ketamine 50 mg/kg, xylazine 10 mg/kg body weight, ip) and were perfused transcardially with 10 ml 0.01 M PBS pH 7.4, followed sequentially by 10 ml of 4% paraformaldehyde in Na-acetate buffer, pH 6.0, and then by 50 ml of 4% paraformaldehyde in Borax buffer, pH 8.5. The brains were rapidly removed and stored in 4% paraformaldehyde in 0.1 M phosphate buffer (PB), pH 7.4, overnight at 4°C. Serial 25 μm thick coronal sections were cut on a Leica VT 1000S vibratome through the preoptic area. The cortical regions were removed from the sections of wild type animals to be able to distinguish among the sections of wild type and nNOS^−/−^ animals. Then the wild type and nNOS^−/−^ sections were processed together.

The sections were treated with 0.5% H_2_O_2_ in PBS for 15 min, cryoprotected in 15% sucrose in PBS for 15 min at room temperature and in 30% sucrose in PBS overnight at 4°C, and then quickly frozen over liquid nitrogen and thawed. The freezing-thawing cycle was repeated three times to improve the antibody penetration.

The pretreated sections were incubated with 10% normal horse serum for 20 min, and then were placed in a mixture of rabbit anti-nNOS serum (1:200; #617000; Thermo Fisher) and guinea pig anti-GnRH serum (1:30,000) for 4 days at 4°C. After rinsing in PBS and 0.1% cold water fish gelatin/1% bovine serum albumin (BSA) in PBS, the sections were incubated in donkey anti-rabbit IgG conjugated with 0.8 nm colloidal gold (Electron Microscopy Sciences, Fort Washington, PA, USA) diluted at 1:100 and biotinylated donkey anti-guinea pig IgG diluted at 1:500 in PBS containing 0.1% cold water fish gelatin and 1% BSA overnight at 4°C. After washing in PBS, the sections were fixed in 1.25% glutaraldehyde in 0.1 M PB for 10 min at room temperature. After further rinsing in PBS the sections were washed in Aurion ECS buffer (Aurion; 1:10) diluted in distillated water (DW). The sections were rinsed in 0.2 M sodium citrate pH 7.5, and then the gold particles were silver intensified with the Aurion R-Gent SE-LM Kit. The sections were placed in 0.05% gold-chloride for 2 × 5 min at room temperature, washed in 0.2 M sodium citrate, pH 7.5, and in 3% sodium-tiosulfate solution for 10 min each at room temperature. Then the sections were treated in avidin-biotin-peroxidase complex (ABC Elite 1:100) and the GnRH-immunoreactivity (IR) was developed in 0.05% DAB/0.005% H_2_O_2_ in 0.05 M Tris buffer, pH 7.6.

Sections were osmicated for 30 min at room temperature, and then treated with 2% uranyl acetate in 70% ethanol for 30 min. Following dehydration in an ascending series of ethanol and acetonitrile (Sigma), the sections were flat embedded in Durcupan ACM epoxy resin (Fluka) on liquid release agent (Electron Microscopy Sciences)-coated slides, and polymerized at 56°C for 2 days. After polymerization, 60–70 nm thick ultrathin sections were cut with Leica UCT ultramicrotome (Leica Microsystems). The ultrathin sections were mounted onto Formvar-coated, single slot grids, and examined with a JEOL-100 C transmission electron microscope.

The specificity of the antisera were described earlier: rabbit antiserum against nNOS (Szabadits et al., 2007) and guinea pig antiserum against GnRH (Hrabovszky et al., 2011).

### Real-Time PCR Detection of Glp1r and Nos1 in GnRH Neurons

Harvesting single-cell mRNAs using patch pipettes is a well-established way to collect mRNA for RT-PCR from neurons, including GnRH neurons in various laboratories (Pape et al., 2001; Xu et al., 2008; Zhang et al., 2009, 2015; Tanaka et al., 2010; Bhattarai et al., 2011). In our present study, the mRNA content of the individual GnRH neurons of male mice used in PCR experiments was also harvested using the patch clamp pipette, as described earlier (Farkas et al., 2013; Vastagh et al., 2015) with slight modification. Patch pipettes were pulled from capillaries sterilized at 180°C for 6 h and filled with autoclaved intracellular pipette solution containing (in mM) HEPES 10, K-gluconate 130, KCl 10, NaCl 10, EGTA 1 and MgCl_2_ 0.1 (pH 7.3 with KOH, osmolarity adjusted to 300 mOsm with D-sorbitol). The resistance of the patch electrodes was 2–3 MΩ. The RNA samples were collected from GnRH-GFP neurons of acute brain slices kept in oxygenated aCSF at 33°C (Farkas et al., 2013; Vastagh et al., 2015) using extra care to avoid any glial RNA contamination with the protocol suggested by Fuzik et al. (2016). The collected cytoplasm was reverse transcribed directly in 20 μl reactions using the ViLO SuperScript III cDNA reverse transcription (RT) kit (Thermo Fisher Scientific, Waltham, MA, USA). For negative controls, the intracellular pipette solution was used in the RT reactions. The obtained cDNA served as template for the subsequent pre-amplification using the Preamp Master Mix kit (Thermo Fisher Scientific) according to the manufacturer’s protocol. The pre-amplification products were diluted 1:10 with 0.1× TE buffer before use in qPCR. Real-time PCR was applied using inventoried TaqMan gene expression assays (Thermo Fisher Scientific) as follows: Gnrh1 (assay ID: Mm01315604_m1), Glp1r (Mm00445292_m1), Nos1 (Mm01208059_m1), Gfap (Mm01253033_m1) and a housekeeping gene Gapdh (Mm99999915_g1). Each assay consisted of a FAM dye-labeled TaqMan MGB probe and two primers. Thermal cycling conditions of the qPCR were as follows: 2 min at 50°C and 20 s at 95°C, followed by 40 cycles of 3 s at 95°C and 30 s at 60°C using the ViiA 7 real-time PCR platform (Thermo Fisher Scientific). Each cDNA sample was checked for Gfap mRNA expression and only Gfap-negative samples were used in the analysis.

Due to the low expression of Glp1r, three pooled samples from three mice were used in the RT-PCR experiments. Each pooled sample contained 10 GnRH neurons. In order to investigate Nos1 expression, individual GnRH neurons were separately examined (30 neurons from five animals).

### Statistical Analysis

Each experimental group included minimum 10 neurons from six to seven animals in the electrophysiological measurements. Recordings were stored and analyzed off-line. Event detection was performed using the Clampfit module of the PClamp 10.4 software (Molecular Devices).

Mean firing rate and mPSC frequency were calculated as number of spikes divided by the length of the respective period (5 min and 10 min, respectively). Bursts were defined according to Lee et al. (2012). Burst frequency was calculated by dividing the number of bursts with the length of the respective time period. Intraburst frequency was calculated by dividing the number of spikes with the length of the respective burst. Percentage changes resulted from Exendin-4 application were calculated by dividing the value to be analyzed before (5 min) and after (the subsequent 10 min) Exendin-4 administration.

Group data were expressed as mean ± standard error (SEM). Statistical significance was analyzed using the ANOVA followed by Newman-Keuls (NK) *post hoc* test (GraphPad Software Inc., La Jolla, CA, USA), and considered at *p* < 0.05 (i.e., 95% confidence interval).

## Results

### Exendin-4 Increases Firing Rate of GnRH Neurons

**Figure 1:**
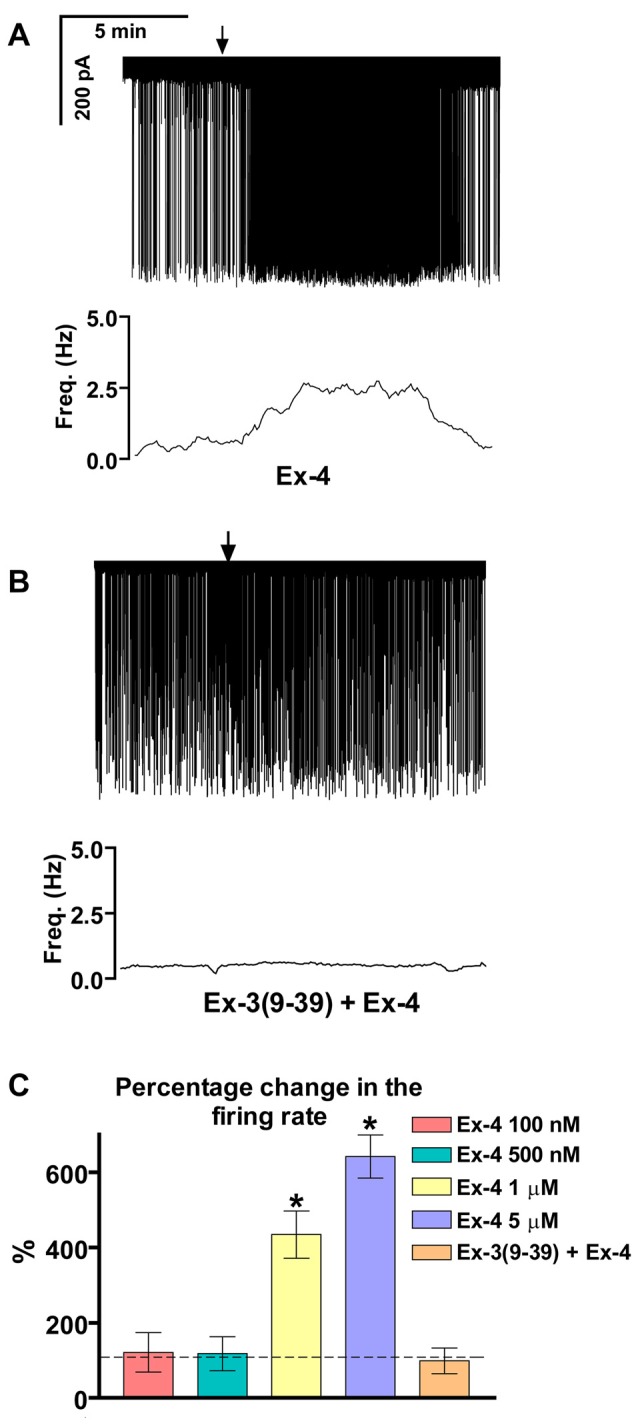
**Effect of glucagon-like peptide-1 (GLP-1) agonist Exendin-4 on the firing of gonadotropin-releasing hormone (GnRH) neurons in brain slices of male mice. (A)** 1 μM Exendin-4 increases the firing rate in GnRH neurons in 5 min. The frequency distribution graph under the recording also shows this profound change. **(B)** Pretreatment of the brain slice with the GLP-1 receptor (GLP-1R) antagonist Exendin-3(9–39) (1 μM) inhibits the effect of Exendin-4 on firing. The frequency distribution graph under the recording confirms this observation. **(C)** Bar graph shows the significant change in the firing rate at 1 and 5 μM, but not at 100 and 500 nM. The graph also demonstrates elimination of the effect upon pretreatment the brain slice with the GLP-1R antagonist. Arrows show application of Exendin-4. *p < 0.05.

Loose patch recordings revealed that silent (spontaneously not firing) GnRH neurons (approx. 25% of all GnRH neurons) could not be activated by Ex-4 administration and therefore, they were discarded from the subsequent analysis. All of the firing GnRH neurons recorded were burst-type neurons and in these neurons Exendin-4 (1 μM; Eng et al., 1992; Raufman et al., 1992; Acuna-Goycolea and van den Pol, 2004) increased the mean firing rate to 434 ± 69.9% of the control (*N* = 10; ANOVA + NK; *p* < 0.05; Figure 1A, Table 1). Frequency distribution graph shows the time course of firing rate. Note that the effect of Exendin-4 is washed out in 10 min (Figure 1A). The average number of spikes within a burst increased to 162 ± 32.9% (from 3.2 ± 0.2 to 5.2 ± 0.2, ANOVA + NK; *p* < 0.05), burst frequency increased to 381 ± 65.2% (from 0.07 ± 0.03 Hz to 0.26 ± 0.02 Hz, ANOVA + NK; *p* < 0.05), and intraburst frequency increased to 172 ± 54.7% (from 6.2 ± 0.7 Hz to 10.7 ± 0.5 Hz; ANOVA + NK; *p* < 0.05) of the control. Lower concentrations of Exendin-4 (100–500 nM) caused no significant change in the average firing rate (Figure 1C, Table 1). In contrast, a higher dose (5 μM) evoked a robust increase in the firing rate (Figure 1C, Table 1). Dose-response curve (Figure 1C) reveals that Exendin-4 is ineffective at 100–500 nM, but causes profound increase in the firing rate at 1–5 μM. Therefore, the 1 μM concentration was used in all subsequent experiments. Of note, this concentration is in a good accordance with the dose used by other laboratories on other types of hypothalamic neurons (Acuna-Goycolea and van den Pol, 2004). When the brain slice was pretreated with the GLP-1R antagonist Exendin-3(9–39) (1 μM), no alteration in the basal firing rate was observed (Table 2). Then, this pretreatment fully eliminated the effect of Exendin-4, the mean firing rate showed no change (98 ± 38.1%; *N* = 10; Figure 1B, Table 2). Burst parameters showed no change either. Bar graph summarizes the percentage changes in the mean firing rate as a result of Exendin-4 application (Figure 1C), demonstrating that Exendin-4 significantly increased mean firing rate and the effect could be abolished by pretreatment with the specific GLP-1R antagonist.

**Table 1:** **Changes in firing rate in gonadotropin-releasing hormone (GnRH) neurons upon Exendin-4 administration at various concentrations of this agonist**.

	Basal firing rate (Hz)	After agonist Ex-4	
		in Hz	in %
Ex-4 (100 nM)	0.57 ± 0.19	0.68 ± 0.22	121 ± 52.6
Ex-4 (500 nM)	0.50 ± 0.27	0.59 ± 0.23	118 ± 45.3
Ex-4 (1 μM)	0.52 ± 0.23	2.25 ± 0.18	434 ± 69.9*
Ex-4 (5 μM)	0.61 ± 0.31	3.91 ± 0.22	642 ± 57.1*

The first column contains firing rate before any drug administration (basal firing rate), the second and third columns provide change in Hz and percentage in firing rate after the single bolus agonist administration. *p < 0.05.

**Table 2:** **Changes in firing rate in GnRH neurons upon Exendin-4 administration with antagonist (Exendin3(9–39)) pretreatment**.

	Basal firing rate (Hz)	During antagonist Ex3(9–39) (Hz)	After agonist Ex-4	
			in Hz	in %
Ex3(9–39)	0.56 ± 0.22	0.58 ± 0.32	0.57 ± 0.29	98 ± 38.1

*The first column contains firing rate before any drug administration (basal firing rate), the second column shows firing rate during antagonist pretreatment, the third and fourth columns provide Hz and percentage change in firing rate after the single bolus agonist administration*.

### Effect of Exendin-4 on the GABAergic mPSCs of GnRH Neurons

**Figure 2:**
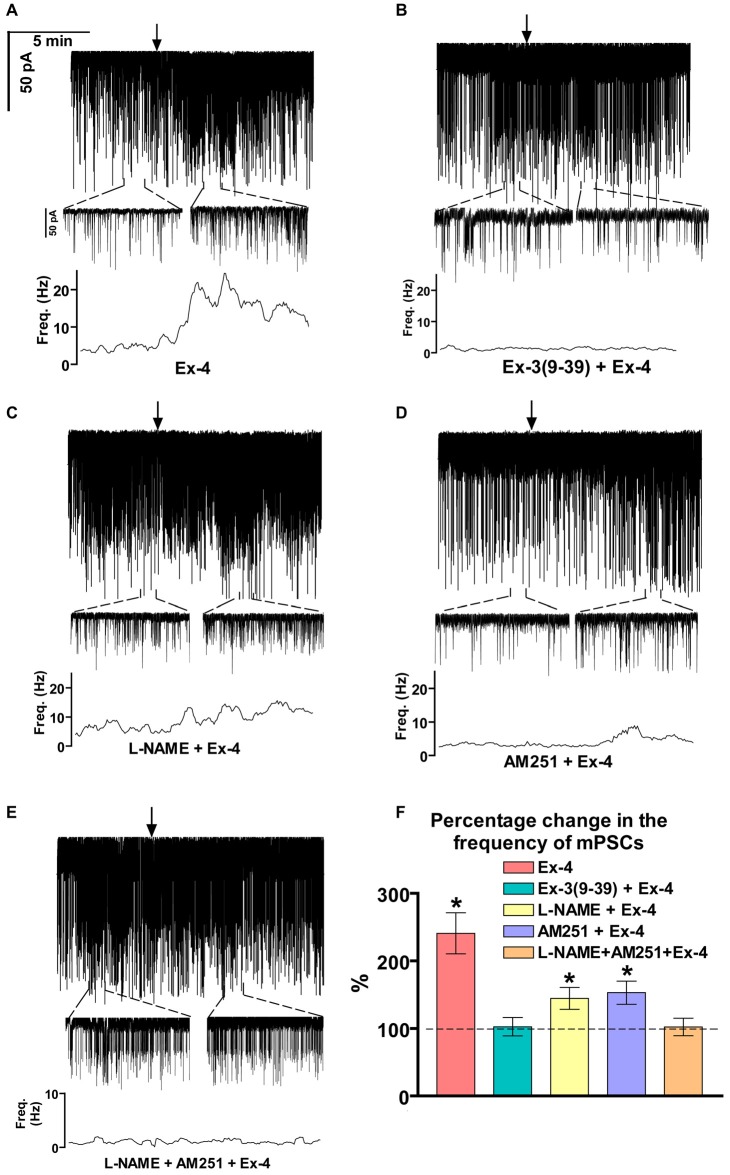
**Effect of Exendin-4 (1 μM) on the miniature postsynaptic currents (mPSCs) in GnRH neurons of male mice. (A)** Exendin-4 increased the frequency of the mPSCs with no change in the average amplitude. One-minute-long periods of the recording before and after application of Exendin-4 are drawn under the recording. The time course of frequency depicted under the zoomed periods shows change in the frequency. **(B)** Effect of Exendin-4 on the mPSCs was abolished by the pretreatment with Exendin-3(9–39). The zoomed periods and the frequency distribution graph confirm this observation. **(C)** Effect of Exendin-4 was eliminated only partially when the slice was pretreated with the nitric oxide-synthase (NOS) inhibitor Nω-Nitro-L-arginine methyl ester hydrochloride (L-NAME). **(D)** Similar partial inhibition was observed in the case of pretreatment with cannabinoid receptor type-1 (CB1) antagonist 1-(2, 4-dichlorophenyl)-5-(4-iodophenyl)-4-methyl-N-(1-piperidyl)pyrazole-3-carboxamide (AM251). **(E)** Full blockade could be accomplished by simultaneous blocking of the NO- and endocannabinoid signaling mechanisms. **(F)** Bar graph reveals that Exendin-4 significantly elevated the frequency of mPSCs. Full inhibition could be achieved by antagonizing the GLP-1R, whereas blockade of either the NO or the endocannabinoid system results in partial inhibition only. Arrows show the onset of Exendin-4 administration. *p < 0.05.

Positive correlation between the firing rate and the frequency of GABAergic mPSCs in GnRH neurons has been well established (Chu and Moenter, 2005; Christian and Moenter, 2007; Farkas et al., 2010, 2013). Since Exendin-4 increased firing rate, we investigated further the effect of Exendin-4 by examining its action on the mPSCs. The administration of Exendin-4 (1 μM) resulted in a significant increase in the mean mPSC frequency (Figure 2A, Table 3) in all GnRH neurons studied, reaching 240.7 ± 30.42% of control values (*N* = 10; ANOVA + NK; *p* < 0.05). The magnified periods and the distribution graph of the frequency depicted under the recording also demonstrated an elevated mPSC frequency, resulting from Exendin-4 administration. Distribution graph revealed that the frequency can reach a peak value as high as 20 Hz in individual GnRH neurons following Exendin-4 application. Elevation of the frequency started to disappear after 10 min washout period. Amplitude of the mPSCs, however, showed no significant alteration (107.5 ± 14.2%; Table 5). Rise and decay time constants of the individual mPSCs showed no modification, either (rise: 97.7 ± 21.9%, from 6.18 ± 1.27 to 5.99 ± 1.39 ms; decay: 92.9 ± 18.1%, from 26 ± 2.4 to 24 ± 3.2 ms). Pretreatment of the brain slice with the antagonist Exendin-3(9–39) caused no change in the basal mPSC frequency, but abolished the Exendin-4 evoked increase in this parameter (Figure 2B, Tables 4, 6) providing evidence for the involvement of the GLP-1R in the effect (102.4 ± 13.6%; *N* = 11, ANOVA + NK; *p* > 0.05). This observation was confirmed by the zoomed periods under the recording and the frequency distribution graph, respectively.

**Table 3:** **Changes in miniature postsynaptic currents (mPSC) frequency upon application of Exendin-4 or L-arginine**.

	Basal frequency (Hz)	Change after Ex-4 or L-arginine	
		in Hz	in %
Ex-4	2.42 ± 0.26	5.81 ± 0.28	240.7 ± 30.42*
L-arginine	2.26 ± 0.29	3.71 ± 0.31	164 ± 15.1*

The first column contains mPSC frequency before any drug administration (basal frequency), the second and third columns show frequency in Hz and % after single bolus Exendin-4 or L-arginine administration. *p < 0.05.

**Table 4:** **Changes in mPSC frequency upon application of Exendin-4 in the presence of various blockers**.

	Basal frequency (Hz)	During antagonists and inhibitors (Hz)	Change after Ex-4	
			in Hz	in %
Ex3(9–39)	2.22 ± 0.28	2.31 ± 0.37	2.35 ± 0.14	102.4 ± 13.6
L-NAME	2.50 ± 0.26	2.34 ± 0.49	3.37 ± 0.34	144.5 ± 16.06*
AM251	2.28 ± 0.23	3.19 ± 0.37	4.88 ± 0.32	153.1 ± 17.14*
L-NAME + AM251	2.26 ± 0.24	3.17 ± 0.32	3.23 ± 0.28	102.2 ± 12.8
GDP-βS	2.34 ± 0.23	2.30 ± 0.44	2.48 ± 0.18	108.0 ± 12.0
CPTIO	2.31 ± 0.26	2.44 ± 0.36	3.73 ± 0.32	153.3 ± 20.06*
intraNPLA + AM251	2.25 ± 0.22	3.20 ± 0.29	3.35 ± 0.27	104.8 ± 6.15
L-NAME + AMG9810	2.42 ± 0.27	2.32 ± 0.29	2.46 ± 0.32	106.0 ± 9.87
NPLA + PF3845	2.18 ± 0.22	3.10 ± 0.32	3.13 ± 0.29	101.0 ± 4.36

The first column contains mPSC frequency before any drug administration (basal frequency), the second column shows frequency after various inhibitor pretreatments, the third and fourth columns provide frequency change in Hz and percentage after single bolus Exendin-4 administration. *p < 0.05.

**Table 5:** **Changes in mPSC amplitude on GnRH neurons upon application of Exendin-4 or L-arginine**.

	Basal amplitude (pA)	Change after Ex-4 or L-arginine	
		in pA	in %
Ex-4	38 ± 5.3	40 ± 5.6	107.5 ± 14.2
L-arginine	45 ± 4.7	43 ± 4.9	96 ± 10.6

**Table 6:** **Changes in mPSC amplitude on GnRH neurons upon application of Exendin-4 after pretreatment with various antagonists or inhibitors**.

	Basal amplitude (pA)	During antagonists and inhibitors (pA)	Change after Ex-4	
			in pA	in %
Ex3(9–39)	41 ± 6.2	39 ± 4.8	37 ± 5.2	96 ± 11.5
L-NAME	35 ± 7.6	37 ± 6.3	37 ± 5.3	102 ± 12.2
AM251	32 ± 8.8	37 ± 6.4	37 ± 4.8	100 ± 11.6
L-NAME + AM251	39 ± 6.7	35 ± 7.2	35 ± 5.0	99 ± 8.7
GDP- βS	35 ± 6.1	36 ± 4.9	33 ± 5.7	93 ± 9.3
CPTIO	42 ± 5.5	38 ± 5.9	41 ± 6.1	110 ± 15.4
intraNPLA + AM251	43 ± 7.0	39 ± 5.7	41 ± 4.9	106 ± 12.3
L-NAME + AMG9810	43 ± 7.0	39 ± 5.7	41 ± 6.3	106 ± 12.3
NPLA + PF3845	40 ± 5.9	42 ± 4.5	40 ± 7.1	95 ± 8.9

### Involvement of NO and 2-Arachidonoylglycerol (2-AG) Signaling Mechanisms

Increase in the frequency of the GABAergic mPSCs could be evoked by activation of the NO machinery in hypothalamic neurons (Di et al., 2009). Therefore, we examined whether this mechanism was involved in the elevation of mPSC frequency after Exendin-4 application in GnRH neurons. In order to block NOS, the slices were pretreated with L-NAME (100 μM). This pretreatment caused no alteration in the basal mPSC frequency and amplitude (Tables 4, 6). Recording of the mPSCs showed, however, that in the presence of L-NAME Exendin-4 was still able to increase the frequency of mPSCs to 144.5 ± 16.06% of the value measured prior to Exendin-4 application without affecting the amplitude (Figure 2C, Tables 4, 6). This percentage value was, however, significantly lower (*N* = 12, ANOVA + NK; *p* < 0.05) than the one observed in the absence of L-NAME. Nevertheless, full elimination of the Exendin-4 effect could not be achieved. Both the zoomed periods and the frequency distribution graph under the recording also revealed this partial attenuation. The percentage elevation differed not only from the change observed in the absence of L-NAME, but also from the value when Exendin-4 was administered in the presence of GLP-1R antagonist (*p* = <0.05).

Our earlier works suggested that tonic 2-AG release could influence synaptic transmission to GnRH neurons (Farkas et al., 2010). In order to examine whether modulation of this tonic endocannabinoid release was also involved in the effect of Exendin-4, the CB1 inverse agonist AM251 (1 μM) was applied to the slice. In accordance with our earlier results, (Farkas et al., 2010), blockade of the retrograde endocannabinoid signaling machinery elevated the basal mPSC frequency without affecting the amplitude (Tables 4, 6). Nevertheless, it decreased the effect of Exendin-4 (Figure 2D, Tables 4, 6). The frequency of mPSCs was raised by Exendin-4 to 153.1 ± 17.14% of the value recorded before Exendin-4 application (Tables 4, 6). This percentage increase is significantly lower (*N* = 11: ANOVA + NK; *p* < 0.05) than the one measured in the absence of AM251. However, similarly to the inhibition of the NO-release, administration of AM251 did not fully eliminate the action of Exendin-4. Both the magnified periods and the frequency distribution revealed that elevation of the frequency of mPSCs also differed significantly from the one measured in the presence of GLP-1R antagonist (*p* < 0.05).

Blocking either NO-production or the presynaptic CB1 inhibited the effects of Exendin-4 only partially. Therefore, we examined whether simultaneous blockade of both signaling mechanisms could eliminate the Exendin-4 triggered changes in the mPSC frequency. The presence of both AM251 and L-NAME in the aCSF fully abolished the effect of Exendin-4 (102.2 ± 12.8%; *N* = 10; Figure 2E, Tables 4, 6). The zoomed recordings and the frequency distribution also confirmed this result. Thus, activation of the GLP-1R resulted in no significant modification in the frequency of the mPSCs, suggesting simultaneous participation of both NO and endocannabinoid retrograde signaling mechanisms.

Bar graph summarizes the effect of Exendin-4 on the mean frequency of the mPSCs and full inhibition of the Exendin-4 triggered action by antagonizing GLP-1R. The graph also depicts that blocking NO-synthesis and CB1 resulted in partial elimination of the action of Exendin-4, whereas blockade of both mechanisms abolished the Exendin-4 effect (Figure 2F).

### Effect of Exendin-4 on GABAergic mPSCs is Direct on GnRH Neurons via Activation of NO-Production in the Recorded Neurons

**Figure 3:**
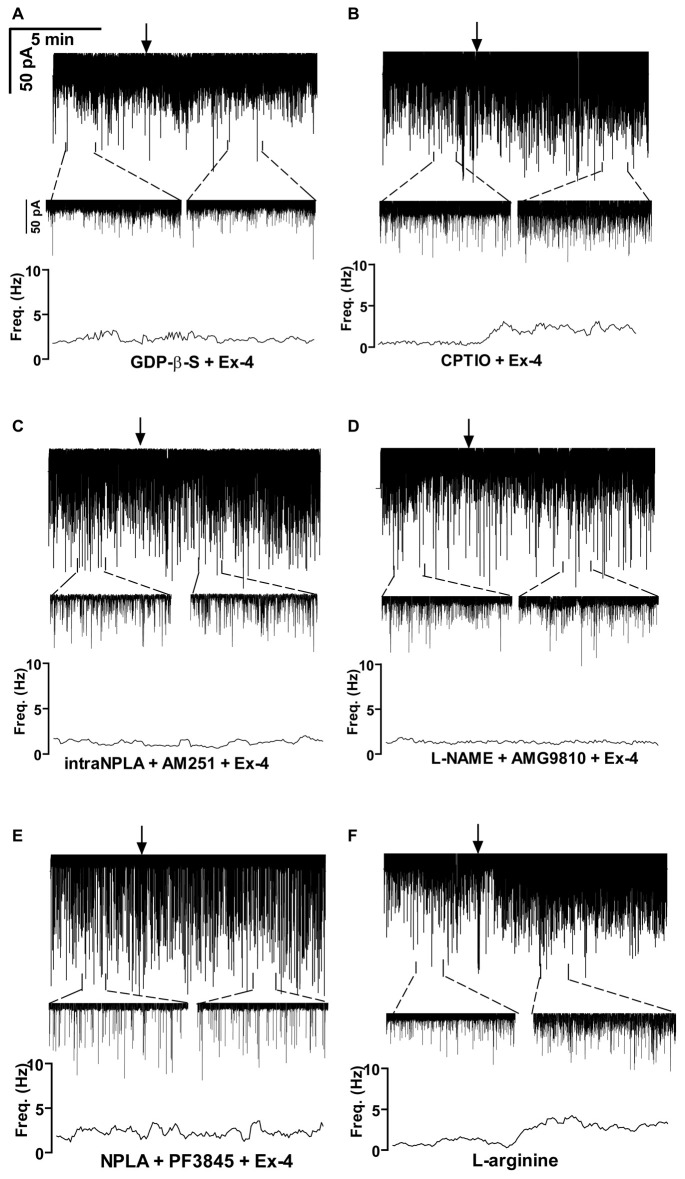
**Exendin-4 acts directly on GnRH neurons by activating a retrograde NO- and an anandamide-transient receptor potential vanilloid 1 (TRPV1) signaling mechanism. (A)** Exendin-4 was unable to modify frequency of mPSCs when G-proteins were intracellularly blocked by GDP-β-S in the recorded GnRH neuron. **(B)** Partial inhibition was observed when the NO-scavenger 2-(4-carboxyphenyl)-4,4,5,5-tetramethylimidazoline-1-oxyl-3-oxide (CPTIO) was applied intracellularly in the GnRH neuron. **(C)** Full inhibition was achieved when neuronal NO synthase (nNOS) was inhibited by the intracellularly applied N^5^-[Imino(propylamino)methyl]-L-ornithine hydrochloride (NPLA) and the endocannabinoid pathway was blocked by AM251. **(D)** Simultaneous blocking of NOS by L-NAME and intracellular inhibition of the TRPV1 receptor by 2E-N-(2,3-Dihydro-1,4-benzodioxin-6-yl)-3-[4-(1,1-dimethylethyl)phenyl]-2-Propenamide (AMG9810) in the GnRH neuron abolished the effect of Exendin-4. **(E)** Inhibition of both nNOS by NPLA and anandamide degradation by PF3845 resulted in full elimination of action of Exendin-4. **(F)** The NO-donor L-arginine elevated frequency of the mPSCs. Arrows show the onset of Exendin-4 or L-arginine administration.

GLP-1R is a member of the G-protein coupled receptor (GPCR) family, and an intracellularly applied G-protein blocker GDP-β-S is supposed to inhibit its function in the recorded GnRH neuron exclusively, without affecting cells in the vicinity of the GnRH neuron. In order to prove the direct action of Exendin-4 in GnRH neurons, its effect on the mPSCs was further examined in the intracellular presence of the GDP-β-S (2 mM). Intracellular application of GDP-β-S caused no change in the basal mPSC frequency and amplitude (Tables 2, 3), but eliminated the effect of the GLP-1R agonist Exendin-4 on the frequency of the mPSCs (108.0 ± 12.0%; *N* = 10; Figure 3A, Tables 4, 6). Both the zoomed periods and the frequency distribution graph support this result.

Earlier in this manuscript we showed the involvement of NO-signaling in the action of Exendin-4. To determine the cellular source of NO, we dissected this regulatory mechanism further by applying the NO-scavenger CPTIO (1 mM) intracellularly in the GnRH neuron. The pretreatment alone exerted no effect on the basal mPSC frequency and amplitude (Tables 4, 6). Under such circumstances, administration of Exendin-4 increased the frequency of the mPSCs to 153.3 ± 20.06% of the value measured before agonist application (*N* = 12; ANOVA + NK; *p* < 0.05), although it was still significantly lower than in the absence of CPTIO (*p* < 0.05; Figure 3B, Tables 4, 6). Magnified periods and the frequency distribution under the recording verified this partial inhibition.

In another experiment the nNOS inhibitor NPLA was applied intracellularly (1 μM) in the extracellular presence of AM251 (1 μM) and then the effect of Exendin-4 was examined on the mPSCs of GnRH neurons. As expected, the pretreatment alone elevated frequency of the mPSCs due to the inhibition of the tonic 2-AG release without affecting the amplitude. Simultaneous application of NPLA and AM251 fully abolished action of Exendin-4 on the frequency of mPSCs (104.8 ± 6.15% of the value before Exendin-4 was added, Figure 3C, Tables 4, 6), verifying further that GnRH neuron was the source of the released NO. The zoomed periods together with the frequency distribution graph also demonstrates the total inhibition.

### The Retrograde 2-AG Pathway is Regulated by Anandamide-TRPV1 Signaling

A recent article has revealed the involvement of the TRPV1 receptor in the inhibition of 2-AG production and in the retrograde endocannabinoid signaling mechanism in hippocampal neurons (Lee et al., 2015). We also examined the putative role of TRPV1 in the decreased tonic 2-AG production in GnRH neurons. Intracellular administration of the TRPV1 antagonist AMG9810 (10 μM) in the presence of L-NAME showed no alteration in the basal mPSC frequency and amplitude, but it completely abolished the effect of Exendin-4 on the frequency of mPSCs in GnRH neurons (106.0 ± 9.87%; *N* = 12; Figure 3D, Tables 4, 6).

Anandamide is an endogenous ligand of TRPV1. Thus, we investigated its role in the activation of TRPV1 by inhibiting FAAH (degrading enzyme of anandamide). The FAAH inhibitor PF3845 (5 μM) was applied intracellularly whereas the NO signaling was blocked by the nNOS inhibitor NPLA (1 μM). Under these conditions, the basal mPSC frequency and amplitude showed no change (Tables 4, 6), but the action of Exendin-4 on the mPSC frequency was fully eliminated in GnRH neurons (101.0 ± 4.36%; *N* = 10; Figure 3E, Tables 4, 6).

In order to confirm further the function of NO in GnRH neurons, the NO-donor L-arginine (1 mM) was applied in the aCSF. Application of L-arginine resulted in elevation of frequency of mPSCs (164 ± 15.1%; *p* < 0.05) with no change in the amplitude (96 ± 10.6%; *N* = 10; Figure 3F, Tables 3, 5).

**Figure 4:**
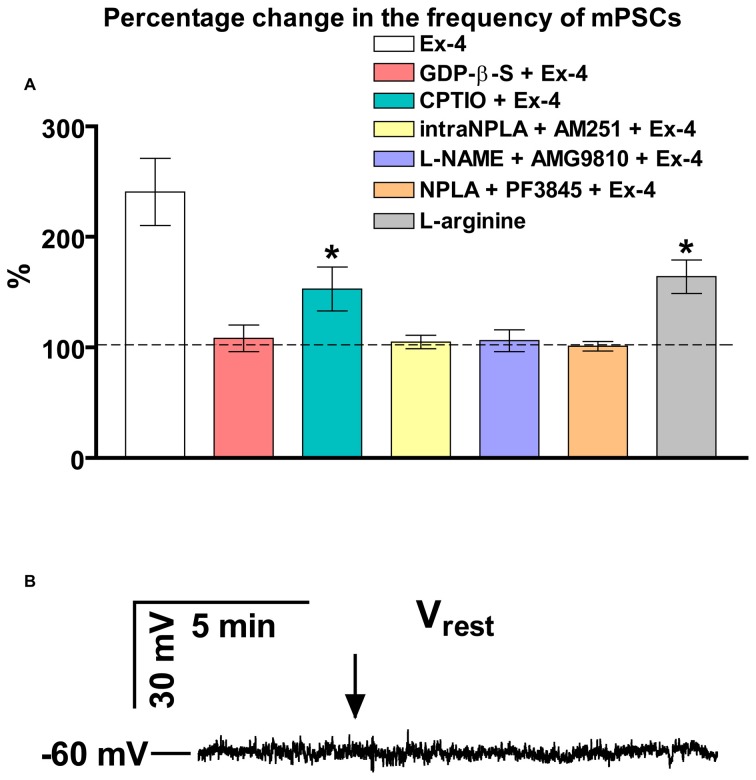
**Summary graph of data demonstrating direct action of Exendin-4 and resting potential measurement. (A)** Bar graph reveals that elevation of the frequency of mPSCs resulted from direct activation of GLP-1R in GnRH neurons. This action activates a NO-signaling machinery and involves an anandamide-TRPV1-coupled mechanism. The Exendin-4 data were taken from Figure 2 for an easier comparability. **(B)** Exendin-4 does not affect resting membrane potential (V_rest_) in GnRH neurons. Arrow shows onset of the Exendin-4 application. *p < 0.05.

Bar graph summarizes these results. Partial inhibition of the action of Exendin-4 was observed when NO-production was eliminated by intracellularly scavenging the produced NO molecules. In contrast, full inhibition was achieved when G-proteins were blocked in the recorded GnRH neurons. Simultaneous inhibition of the nNOS and anandamide-TRPV1 pathways also completely abolished the effect (Figure 4A).

In an additional experiment, effect of Exendin-4 on the resting membrane potential (V_rest_) was examined (Figure 4B). The measurements showed no significant change in this parameter demonstrating that ion channels contributing to the level of V_rest_ are not involved in the process.

### GnRH Neurons are Contacted by GLP-1 Immunoreactive Axons in Mice

**Figure 5:**
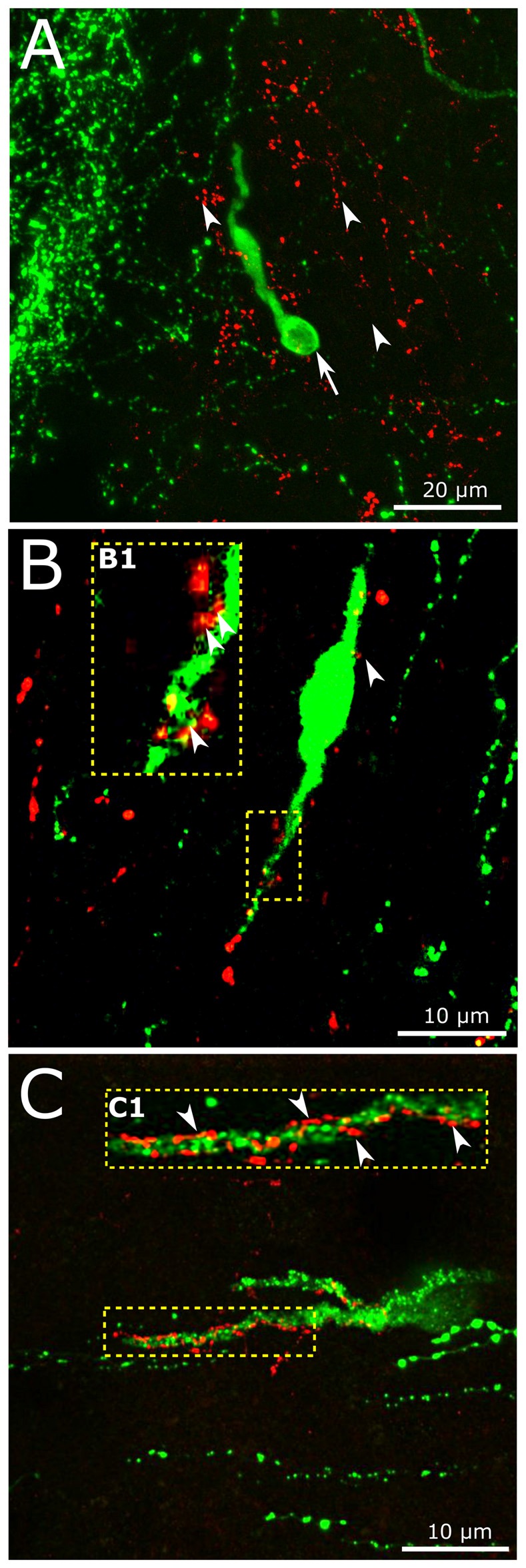
**Interaction of central GLP-1 and GnRH neuronal systems. (A)** Red GLP-1-immunoreactive (IR) axons (arrowheads) and a green GnRH-IR neuron (arrows) appear in the vicinity of the vascular organ of lamina terminalis (OVLT) of the mouse brain. **(B,C)** GLP-1-IR axons establish contacts with the dendrites (enframed by dotted line) of a subset of GnRH neurons exhibiting smooth **(B)** or rough **(C)** surface. The multiple contacts (arrowheads) are shown at higher power in insets **(B1,C1)**.

In accordance with previous data (Larsen et al., 1997; Sarkar et al., 2003; Llewellyn-Smith et al., 2011), we have now detected dense network of GLP-1 IR fibers of the hypothalamic dorsomedial and paraventricular nuclei, the periventricular region of the third ventricle (not shown), the region of the vascular OVLT and the preoptic area where the majority of GnRH neurons reside. The putative involvement of the brain-born GLP-1 in the central regulation of reproduction was studied by the simultaneous detection of the GnRH and GLP-1-IR neuronal systems. Individual GnRH-synthesizing neurons scattered within the network of GLP-1-IR axons (Figure 5A). GLP-1-IR axons approached and established single or occasionally, multiple contacts with one tenth of GnRH neurons. Distribution of the contacts was rather inhomogeneous and exhibited the highest density in the proximity of the OVLT. GLP-1 inputs often targeted the dendritic compartment of GnRH cells (Figures 5B,C). Both the smooth surfaced (Figure 5B) and the rough surfaced (Figure 5C) subtypes of GnRH neurons (Liposits et al., 1984; Witkin and Demasio, 1990) received GLP-1-IR axons. The insets (B1,C1) in Figures 5B,C demonstrate at high power an example for the multiple axo-dendritic contacts established by GLP-1-IR axons on the interacting GnRH neurons in confocal images.

### Double-Labeling Immuno-Electron Microscopy Demonstrates Presence of nNOS in GnRH Neurons

**Figure 6:**
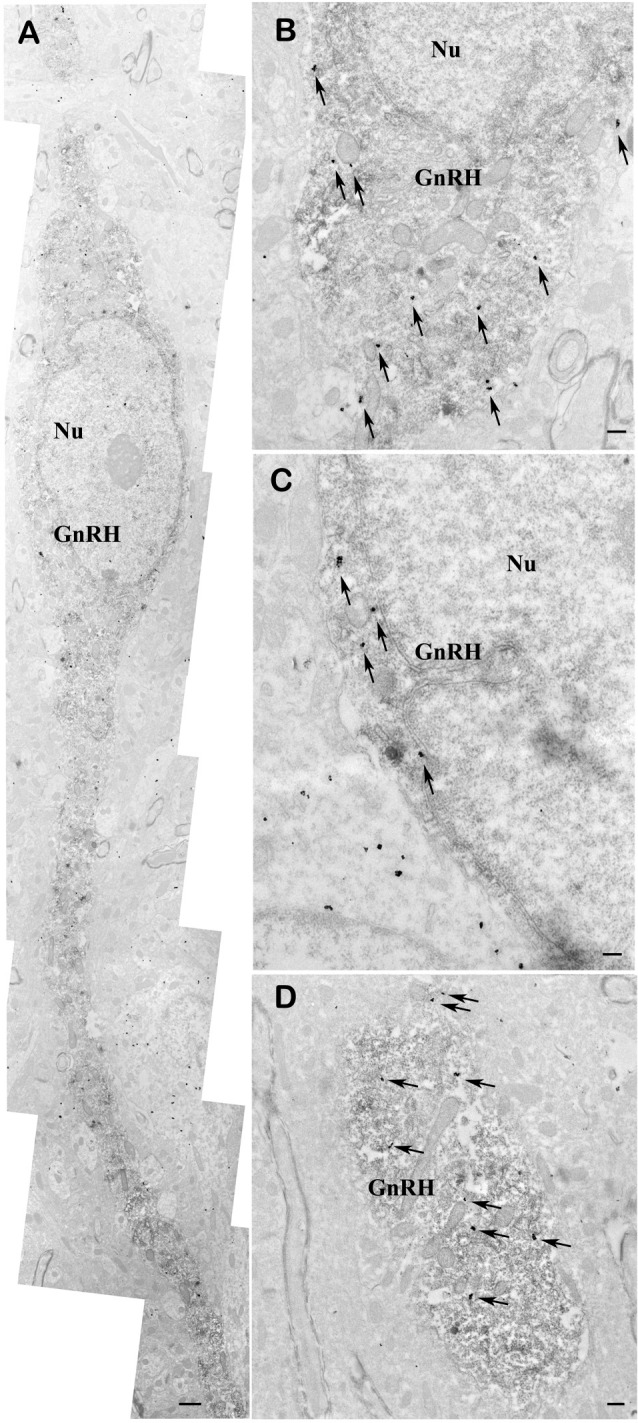
**Co-localization of nNOS and GnRH in the preoptic area of mice.** Electron micrographs showing strong nNOS immunogold labeling in GnRH neurons **(A–D)**. The nNOS-immunoreactivity (nNOS-IR) is detected with highly electron dense gold–silver particles (arrows), while the presence of the electron dense DAB chromogen identifies the GnRH-IR structures. nNOS-IR characterizes both perikarya **(A–C)** and dendrites **(A,D)** of GnRH neurons. Low-power micrograph **(A)** illustrates the overall presence of nNOS-IR in the perikaryon and processes of a fusiform GnRH neuron. High-power micrographs **(B–D)** depict the localization of nNOS-IR in the perinuclear cytoplasm **(B,C)** and the dendritic domain **(D)** of immunolabeled GnRH neurons. Note the expression of nNOS in a neuron located left to the double-labeled GnRH cell **(C)**. Scale bar = 1 μm in **(A)** and 0.4 μm in **(B–D)**, Nu, nucleus; GnRH, gonadotropin-releasing hormone neuron.

To demonstrate the presence of nNOS in the GnRH neurons, double-labeling immuno-electron microscopy was performed. A large number of highly electron dense gold-silver particles denoting the nNOS-IR was detected in the GnRH neurons that were recognized on the presence of the electron dense DAB chromogen. nNOS-IR was present in both the perikaryon (Figures 6A–C) and dendrite (Figure 6D) of GnRH neurons. nNOS-IR was also observed in numerous non-GnRH neurons of the preoptic area, including those residing in the close vicinity of the nNOS-positive GnRH neurons (Figure 6C). In contrast to wild-type animals, in transgenic nNOS^−/−^ mice the immuno-electronmicroscopic study could not detect any specific sign for the expression of nNOS in GnRH and non-GnRH neurons (not shown).

### RT-PCR Confirms the Expression of Glp1r and Nos1 Genes in GnRH Neurons of Mice

**Figure 7:**
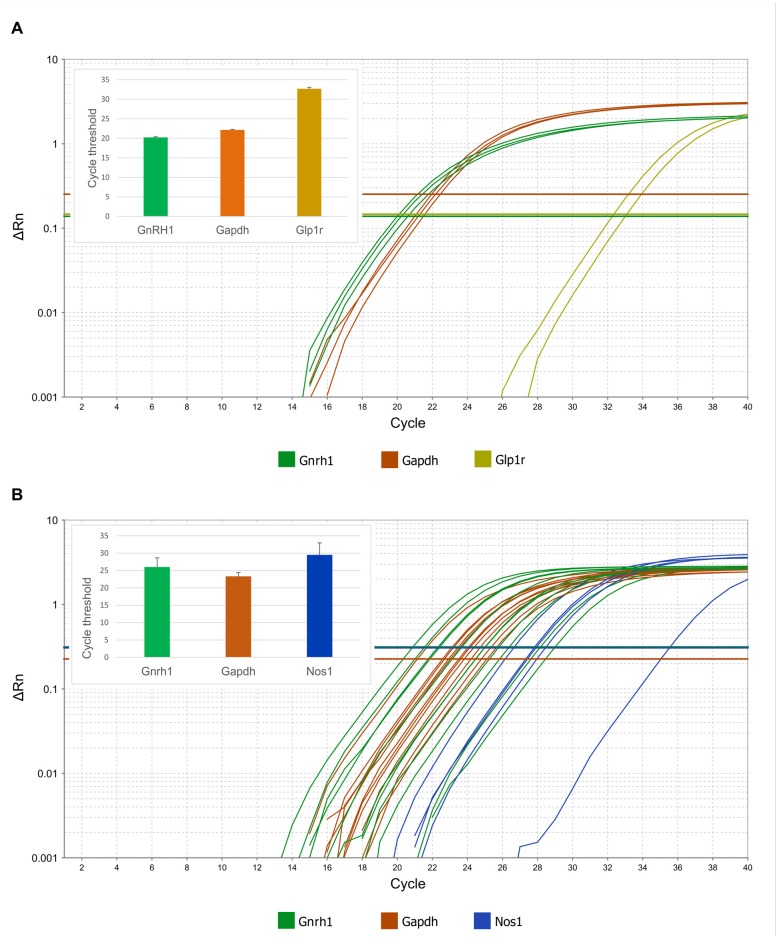
**Expression of Glp1r and Nos1 mRNAs in GnRH neurons harvested by patch electrodes for single-cell qPCR.** Real-time qPCR amplification of Gnrh, Gapdh, Glp1r and Nos1 cDNA from GnRH neurons. **(A)** Expression of Glp1r transcript was detected in two of three pools of GnRH neuronal cytoplasmic samples. The abundance of the Glp1r was low, indicated by its relatively high Ct values (32.5 and 33.1) as compared to the housekeeping gene Gapdh (22.0–22.5). **(B)** Expression of the Nos1 gene in individually harvested Gfap-negative GnRH neuronal samples. The logarithmic scale of Rn and number of PCR cycles are indicated on the Y and X axes, respectively. The onset of the exponential phase (Ct) is indicated by horizontal lines for each target gene. Column charts are in the inserts to show quantitative results of the qPCR experiments.

In addition to Gnrh1 mRNA, expression of Glp1r mRNA was detected in pooled, patch pipette-harvested GnRH-GFP neuron cytoplasm samples at cycle threshold (Ct) 32.7 ± 0.4. The Ct value of Gapdh was 22.3 ± 0.1 (Figure 7A). None of the transcripts were detected in the negative control samples.

Expression of Gnrh1 mRNAs was also verified in each patch pipette-harvested individual GnRH-GFP cytoplasm sample at Ct value of 24.5 ± 0.8. We monitored the samples for potential glial contamination by detecting Gfap mRNA. Samples with any Gfap mRNA expression were omitted from the study. Amplification curves showed that GnRH neurons expressed Nos1 mRNA (Ct 29.5 ± 1.5; Figure 7B). Presence of Nos1 transcript was detected in 5 out of 11 GnRH cytoplasmic samples. The cycle threshold value of the housekeeping gene Gapdh was 23.3 ± 0.3.

## Discussion

Earlier studies described the modulatory effect of GLP-1 on reproduction. In our present work we have provided compelling evidences for the direct regulatory action of GLP-1 on GnRH neurons. Accordingly: (1) GLP-1 excites firing and increases frequency of GABAergic mPSCs in GnRH neurons via GLP-1R; (2) downstream events of GLP-1R involve two retrograde signaling pathways: activation of NO- and suppression of 2-AG signaling mechanisms; (3) suppression of 2-AG pathway is mediated via anandamide-TRPV1 signaling.

### GLP-1 Is Excitatory to GnRH Neurons via GLP-1R

Our results demonstrated that GLP-1 agonist exerts excitatory effect in GnRH neurons. Both firing rate and frequency of GABAergic mPSCs increased upon Exendin-4 administration. Elevation in both firing rate and mPSC frequency correlates well because GABA is excitatory via GABA_A_-R in GnRH neurons (Sullivan et al., 2003; Farkas et al., 2010), although we have to note that GABA is inhibitory via GABA_B_-R (Herbison and Moenter, 2011; Liu and Herbison, 2011). Our data are in accordance with a recent investigation showing stimulatory effect of GLP-1 in neurons of the paraventricular nucleus, the bed nucleus of the stria terminalis and the hippocampus (Cork et al., 2015). Excitatory role of GLP-1 on the reproductive axis has already been suggested, too (Beak et al., 1998; MacLusky et al., 2000; Outeiriño-Iglesias et al., 2015). Our finding is also supported by other studies showing increased spike and mPSC frequency in hypothalamic hypocretin/orexin neurons (Acuna-Goycolea and van den Pol, 2004) or revealing elevated *c-fos* level and firing rate in neurons of parabrachial nucleus (Richard et al., 2014). Furthermore, GLP-1 is able to modulate GABA_A_-R-mediated synaptic currents in hippocampus (Korol et al., 2015) providing further confirmation of our results. The Exendin-4 evoked activation of both firing and mPSCs in GnRH neurons could be antagonized by GLP-1R antagonist suggesting a pivotal, direct role of this receptor in the action. In addition, expression of GLP-1R mRNA was found in the transcriptome of GnRH neurons, indicating the presence of GLP-1R in these cells. Intracellular blockade of GLP-1R, a member of the GPCR family, by GDP-β-S and the subsequent abolishment of the Exendin-4 triggered change in the mPSC frequency also demonstrated existence of functional and active GLP-1R in GnRH neurons. The intracellularly applied NO-scavenger CPTIO, the TRPV1 inhibitor AMG9810 and the FAAH inhibitor PF3845 were effective, too, confirming further the direct action of Exendin-4 in GnRH neurons. Thus, direct effect of GLP-1 agonist on GnRH neurons is revealed beside its earlier suggested indirect action via kisspeptin neurons (Outeiriño-Iglesias et al., 2015).

Although most of the researchers agree that the recorded mPSCs in GnRH neurons are GABAergic under basal conditions, theoretically we cannot exclude that the Exendin-4 induced alteration in the mPSCs is due to the release of glutamate. In our experiments, however, rise and decay time constant (tau) parameters of the PSCs showed no change upon Exendin-4 administration. Since glutamatergic PSCs exhibit significantly shorter tau values than GABAergic ones (Smith and Dudek, 1996), we found no indications for the effect of Exendin-4 on glutamatergic neurotransmission.

One of the putative sources of GLP-1 reaching GnRH neurons was also revealed. GLP-1-IR fibers contacted subset of GnRH neurons in mouse samples showing the assumed role of brain-born GLP-1. Various hypothalamic loci are innervated by GLP-1-containing fibers (Renner et al., 2012; Katsurada et al., 2014) originating from the NST. In this view, it is not surprising that areas where GnRH neurons reside are also innervated by GLP-1-IR axons. The results confirm our hypothesis that GnRH neurons are able to sense metabolic status of the body receiving input signals both directly from the periphery by the gut-born GLP-1 level and indirectly via the brain-born GLP-1 from NST. The multiple sources of the sensed GLP-1 (gut-born via the blood-brain barrier vs. brain-born) can also explain the quasi-contradiction between the ratio of GLP-1 contacted GnRH neurons (10%) and the high proportion of GnRH neurons responding to GLP-1 in the electrophysiological experiments (100%).

### Effect of Exendin-4 on GnRH Neurons is Mediated Partially by Activation of the NO Retrograde Signaling

The electrophysiological results revealed the involvement of the NO retrograde signaling mechanisms in the Exendin-4 evoked action. Inhibition of NOS by L-NAME partially eliminated the rise in the frequency of mPSCs, suggesting that this rise was partially due to an elevated NO level. Scavenging NO intracellularly by the membrane-impermeable CPTIO (Makara et al., 2007) in GnRH neurons provided evidence that the measured GnRH neuron itself was the source of NO in this effect. Involvement of NO-signaling in action of GLP-1 has already been reported at various sites in the brain (Chien et al., 2015; Zhao et al., 2015). Furthermore, our work showing increased mPSC frequency but not mPSC amplitude or rise/decay tau indicates presynaptic excitatory function of the GnRH-produced NO. This has led to the speculation that NO travels as retrograde messenger from the post- to the presynaptic cell (Schuman and Madison, 1991). Similar NO-related retrograde mechanism has already been suggested in the hypothalamus, too (Borgquist et al., 2015).

The actions of NO on reproduction are rather complex. Sodium nitroprusside, a NO-donor, stimulated GnRH release from hypothalamic explants (Moretto et al., 1993). The immortalized, GnRH-producing GT1–7 neuronal cell line expressed nNOS and an enhanced GnRH secretion was observed in these cells when L-arginine, a precursor of NO was administered (Mahachoklertwattana et al., 1994). Leptin-dependent NO signaling in the preoptic region was also shown to facilitate reproduction (Bellefontaine et al., 2014). In contrast, other researchers found suppressive effect of NO on LH secretion (Pinilla et al., 1999). In addition, earlier studies reported absence of nNOS in GnRH neurons (Grossman et al., 1994; Herbison et al., 1996) and that NO inhibited spontaneous firing in adult GnRH neurons (Clasadonte et al., 2008). Our present work revealed the expression of nNOS mRNA and the presence of nNOS protein in mouse GnRH neurons. The high resolution of the used immuno-electron microscopic technique combined with the dual-pH fixation of the samples resulted in the discovery of nNOS protein in mouse GnRH neurons. It can also resolve the discrepancy between our present ultrastructural results and those earlier published light microscopic findings (Grossman et al., 1994; Herbison et al., 1996; Ishihara et al., 2001; Hanchate et al., 2012) that reported the absence of nNOS protein in GnRH neurons. Our electrophysiological investigation provided further proof for NO production of GnRH neurons, which exerts stimulatory action on presynaptic GABAergic axon terminals innervating GnRH neurons. In addition, the NO-donor L-arginine triggered a rise in the mPSC frequency providing further evidence for a functional NO-pathway in GnRH neurons. These data are supported by an earlier article (Varju et al., 2009) also demonstrating expression of nNOS in the GnRH-producing GT1–7 neurons. The discrepancy between our results and those of Clasadonte et al. (2008) can be explained by the different experimental conditions such as recording temperature (room temperature vs. 33°C). Since enzymatic processes (such as the ones involving DGL and nNOS), ion pumps and exchanger fluxes are extremely sensitive to the temperature, this might explain differences. In addition, intracellular (pipette) solution contained low chloride concentration (10 mM) in the work of Clasadonte et al. (2008), whereas we used high-chloride (130 mM) pipette solution in our measurements. Since the physiological chloride content of GnRH neurons is high, the present approach mimics more effectively the physiological condition of GnRH neurons. Nevertheless, although our study reveals that NO released from GnRH neuron as retrograde messenger triggered excitation by acting presynaptically on GABAergic input, it cannot be excluded that NO may elicit inhibition of GnRH neurons when it is anterogradely released from neighboring, non-GnRH neurons and act at postsynaptic sites of GnRH neurons.

### Exendin-4 Acts Partially via Retrograde Endocannabinoid Signaling Pathway in GnRH Neurons

Our study also showed involvement of the retrograde endocannabinoid signaling in the Exendin-4 evoked action. Blockade of CB1 by AM251 resulted in partial inhibition of the Exendin-4 induced effect. In addition, intracellular inhibition of GLP-1R by the G-protein inhibitor GDP-β-S and the resulting total elimination of the effect of Exendin-4 demonstrated that GnRH neuron was the source of not only the NO but also the endocannabinoids mediating the effects of Exendin-4. The presence of the tonic 2-AG retrograde endocannabinoid machinery which can decrease the activity of the GABAergic input to GnRH neurons has already been reported (Farkas et al., 2010). Its role in the mediation of the effect of metabolic signals was also described in various neurons, including GnRH cells (Kola et al., 2008; Farkas et al., 2013). The 2-AG retrograde signaling inhibits GABAergic input to GnRH neurons (Farkas et al., 2010, 2013), however, Exendin-4 triggered excitation in these cells, even when NO signaling was blocked by L-NAME or by the intracellularly applied CPTIO. Therefore, we can deduce that stimulation of GLP-1R results in suppression of the 2-AG pathway. A recent article showed that 2-AG production can be decreased in neurons by activating TRPV1 (Lee et al., 2015). Furthermore, stimulation of GPCRs such as muscarinic acetylcholine receptors or metabotropic glutamate 5 receptors can activate TRPV1 by elevating anandamide level (Maccarrone et al., 2008; Musella et al., 2010). TRPV1 regulates local calcium level below the postsynaptic membrane (Cristino et al., 2006) suppressing activity of DGL and 2-AG production and eventually decreasing GABAergic neurotransmission in striatal neurons (Maccarrone et al., 2008; Musella et al., 2010). These results fit our findings that the GPCR GLP-1R can reduce the tonic 2-AG inhibition of the GABAergic neurotransmission to GnRH neurons through an anandamide-TRPV1 signaling.

A logical question arises why anandamide, which can also bind to CB1, induces no decrease in the mPSC frequency. Theoretically, increased level of anandamide may indeed mimic 2-AG effects and act on presynaptic CB1. However, FAAH (degrading enzyme of anandamide) is postsynaptic, whereas monoacylglycerol lipase (MGL; degrading enzyme of 2-AG) is presynaptic (Gulyas et al., 2004), and brain 2-AG concentration is 170 times higher than anandamide concentration (Stella et al., 1997). Conversely, DGL, the synthesizing enzyme of 2-AG, also occurs postsynaptically in the somato-dendritic domain of neurons, in which FAAH is located (Gulyas et al., 2004; Katona et al., 2006). These data, therefore, do not support the scenario that anandamide could exert a significant direct effect on the presynaptic CB1. Rather, this molecular architecture suggests that instead of acting on the presynaptic CB1, postsynaptic elevation of anandamide level may interfere with the postsynaptic mobilization of 2-AG.

### The NO and Endocannabinoid Retrograde Signaling Pathways are Simultaneously Involved in the Effect of Exendin-4

**Figure 8:**
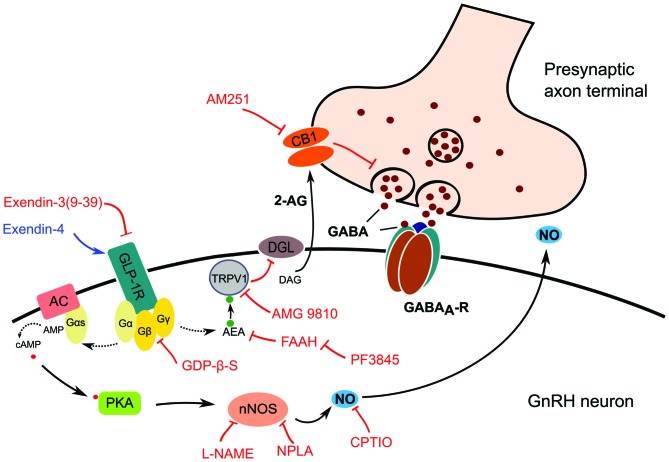
**Schematic illustration of GLP-1R signaling in GnRH neurons and its proposed action on presynaptic GABA release.** Effect of the GLP-1R agonist (Exendin-4) or antagonist (Exendin-3) is mediated by G-protein complex, which may activate two retrograde signaling cascades. The first pathway involves activation of the intracellular TRPV1 which decreases postsynaptic production and release of 2-AG resulting in the suppression of inhibition of the presynaptic GABA release. The second one involves the release of NO produced by nNOS which increases the release probability and vesicular reuptake of GABA at the presynaptic terminal. Abbreviations: GLP-1R, glucagon-like peptide 1 receptor; AC, adenylate cyclase; Gα, Gβ, Gγ, G-protein subunits; DAG, diacylglycerol; DGL, DAG-lipase; CB1, cannabinoid receptor type-1; AM251, CB1 antagonist; 2-AG, 2-arachidonoylglycerol; GABA_A_-R, GABA_A_ receptor; PKA, protein kinase A; nNOS, neuronal nitric oxide synthase; L-NAME, Nω-Nitro-L-arginine methyl ester hydrochloride, a NOS inhibitor; NPLA, *N*^5^-[Imino(propylamino)methyl]-L-ornithine hydrochloride, a nNOS inhibitor; GDP-β-S, GDP-Beta-S trilithium salt, a G-protein inhibitor; CPTIO, Carboxy-PTIO potassium salt, a NO scavenger; TRPV1, transient receptor potential vanilloid 1; AEA, anandamide; AMG9810, TRPV1 antagonist; FAAH, fatty acid amide hydrolase; PF3845, FAAH-inhibitor. Red lines depict inhibitory actions, blue line refers to excitatory action, and dotted lines represent hypothetical relations.

Blockade of either nNOS or CB1 resulted in partial inhibition of the effect of Exendin-4. In contrast, simultaneous blockade of both pathways hindered action of Exendin-4 completely. The results suggest that activation of GLP-1R in GnRH neuron initiates production of NO and suppression of tonic 2-AG as proposed in Figure 8. The schematic diagram describes the proposed mechanism of action of GLP-1 agonist where binding of Exendin-4 to GLP-1R stimulates nNOS via a G-protein mediated pathway. Activated nNOS then results in higher level of NO, which diffuses out from GnRH neuron to stimulate GABA release from the presynaptic terminal. In parallel, binding of Exendin-4 represses synthesis and release of tonic 2-AG via an anandamide-TRPV1-controlled pathway. Suppression of 2-AG in GnRH neurons eventually results in further facilitation of GABA release from the presynaptic terminal into the synaptic cleft. Simultaneous involvement of both NO and endocannabinoid retrograde signaling mechanisms has already been described in the hypothalamus (Di et al., 2009). In their report, Di et al. (2009) found that glucocorticoid suppressed the glutamatergic and facilitated the GABA_A_-R associated GABAergic input to magnocellular neurons of the rat supraoptic nucleus (SON). These glucocorticoid-induced effects were mediated via GPCR and activated retrograde neuronal NO release and endocannabinoid synthesis simultaneously. Nevertheless, the glucocorticoid-related events in the SON are somewhat different from the GLP-1 agonist-triggered ones we found in GnRH neurons because glucocorticoid administration activated both NO and endocannabinoid retrograde mechanisms whereas stimulation of GLP-1R triggered NO release but suppressed 2-AG production. The reason behind this discrepancy might be the distinctive excitatory role of GABA_A_-R in GnRH neurons.

Increase in the frequency of the GABAergic neurotransmission could by itself indicate presence of a presynaptic effect of GLP-1. However, various intracellular blockades of the G-proteins and the cannabinoid and NO pathways in the postsynaptic GnRH neurons inhibited action of GLP-1, excluding this opportunity. Our results, therefore, suggest the direct postsynaptic effect of GLP-1 and activation of the two parallel retrograde signaling mechanisms in GnRH neurons.

The role of GLP-1 in reproduction is rather controversial. Although GLP-1R KO mice are fertile (Preitner et al., 2004), male mice exhibit reduced gonadal weight and female mice show delayed puberty (MacLusky et al., 2000). Human clinical studies and case reports also indicate that GLP-1 plays important roles in the regulation of human fertility (Jeibmann et al., 2005; Fontoura et al., 2014). These observations suggest that although lack of GLP-1 signaling may have no substantial effects on reproduction, this peptide is capable to modulate the reproductive axis. In our present work, we have provided compelling evidence that GLP-1 agonist is able to act directly on GnRH neurons, and this action is mediated by activation of retrograde NO and anandamide-TRPV1-mediated suppression of 2-AG endocannabinoid signaling mechanisms.

Although half-life of endogenous GLP-1 is less than 2 min in the blood-stream (Kieffer et al., 1995) because of its cleavage by dipeptidyl peptidase-4 (DPP-4), recent works have demonstrated that various GLP-1 analogs widely used for treatment of type 2 diabetes and/or obesity are DPP-4 resistant due to their modified molecular structure (Barrera et al., 2009; Katsurada and Yada, 2016). Therefore, these analogs have longer half-life such as several hours. In addition, they can cross the BBB (Hunter and Hölscher, 2012) and remain in the brain for several hours (Secher et al., 2014). In the hypothalamus, fluorescence labeled analogs were observed in the median eminence and the OVLT (Katsurada and Yada, 2016). Thus, our results suggest that GLP-1 agonists might affect regulation of reproduction at the level of GnRH neurons. Furthermore, activation of the NO and anandamide-TRPV1-sensitive endocannabinoid pathways can provide options to fine-tune the reproduction-specific effects of GLP-1.

## Author Contributions

IF designed the experiments, carried out electrophysiology recordings and wrote the manuscript. CV carried out immunohistochemistry and RT-PCR and wrote the manuscript. EF carried out immuno-electron microscopy and wrote the manuscript. FB carried out electrophysiology recordings. KS carried out immunohistochemistry. EH carried out immunohistochemistry and wrote the manuscript. CF designed the immuno-electron microscopy and wrote the manuscript. ZL designed the experiments and wrote the manuscript.

## Conflict of Interest Statement

The authors declare that the research was conducted in the absence of any commercial or financial relationships that could be construed as a potential conflict of interest.
